# RNA-Containing Cytoplasmic Inclusion Bodies in Ciliated Bronchial Epithelium Months to Years after Acute Kawasaki Disease

**DOI:** 10.1371/journal.pone.0001582

**Published:** 2008-02-13

**Authors:** Anne H. Rowley, Susan C. Baker, Stanford T. Shulman, Francesca L. Garcia, Linda M. Fox, Ian M. Kos, Susan E. Crawford, Pierre A. Russo, Rashid Hammadeh, Kei Takahashi, Jan M. Orenstein

**Affiliations:** 1 Department of Pediatrics, Northwestern University Feinberg School of Medicine, The Center for Kawasaki Disease, The Children's Memorial Hospital, Chicago, Illinois, United States of America; 2 Department of Microbiology-Immunology, Northwestern University Feinberg School of Medicine, Chicago, Illinois, United States of America; 3 Department of Pathology, Northwestern University Feinberg School of Medicine, Chicago, Illinois, United States of America; 4 Department of Microbiology and Immunology, Loyola University Stritch School of Medicine, Maywood, Illinois, United States of America; 5 Department of Pathology, Loyola University Stritch School of Medicine, Maywood, Illinois, United States of America; 6 Department of Pathology, Children's Hospital of Philadelphia, Philadelphia, Pennsylvania, United States of America; 7 Department of Pathology, George Washington University School of Medicine, Washington, D. C., United States of America; 8 Department of Pathology, Toho University School of Medicine, Tokyo, Japan; Columbia University, United States of America

## Abstract

**Background:**

Kawasaki Disease (KD) is the most common cause of acquired heart disease in children in developed nations. The KD etiologic agent is unknown but likely to be a ubiquitous microbe that usually causes asymptomatic childhood infection, resulting in KD only in genetically susceptible individuals. KD synthetic antibodies made from prevalent IgA gene sequences in KD arterial tissue detect intracytoplasmic inclusion bodies (ICI) resembling viral ICI in acute KD but not control infant ciliated bronchial epithelium. The prevalence of ICI in late-stage KD fatalities and in older individuals with non-KD illness should be low, unless persistent infection is common.

**Methods and Principal Findings:**

Lung tissue from late-stage KD fatalities and non-infant controls was examined by light microscopy for the presence of ICI. Nucleic acid stains and transmission electron microscopy (TEM) were performed on tissues that were strongly positive for ICI. ICI were present in ciliated bronchial epithelium in 6/7 (86%) late-stage KD fatalities and 7/27 (26%) controls ages 9–84 years (p = 0.01). Nucleic acid stains revealed RNA but not DNA within the ICI. ICI were also identified in lung macrophages in some KD cases. TEM of bronchial epithelium and macrophages from KD cases revealed finely granular homogeneous ICI.

**Significance:**

These findings are consistent with a previously unidentified, ubiquitous RNA virus that forms ICI and can result in persistent infection in bronchial epithelium and macrophages as the etiologic agent of KD.

## Introduction

Kawasaki Disease (KD), the most common cause of acquired heart disease in children in developed nations, is an acute systemic inflammatory illness of young children, affecting the medium-sized arteries, particularly the coronary arteries. KD is manifested by the sudden onset of high-spiking fever, rash, enanthem, exanthem, swelling and redness of the hands and feet, and cervical adenopathy in previously healthy infants and children [1]. Although these findings resolve over 1–2 weeks, 25% of untreated patients develop coronary artery abnormalities; in severe cases, myocardial infarction and sudden death can occur. Although the etiology is unknown, clinical and epidemiologic features are consistent with an ubiquitous infectious agent that usually results in asymptomatic infection but can lead to KD in a very small subset of genetically predisposed individuals [2].

We discovered that oligoclonal IgA plasma cells infiltrate inflamed tissues in acute KD [3]–[5], and made synthetic versions of these oligoclonal antibodies. The KD synthetic antibodies detect antigen in ciliated bronchial epithelium of children who died of KD during the acute illness and in a subset of macrophages in acute stage inflamed KD tissues, including the coronary arteries [6], [7]. We demonstrated that synthetic antibodies derived from immunoglobulin alpha sequences that are more prevalent in acute KD arterial tissue bind more strongly to the antigen in KD ciliated bronchial epithelium than do antibodies derived from immunoglobulin alpha sequences that are less prevalent in acute KD arterial tissue, a characteristic feature of an antigen-driven antibody response [8]. Antigen was not detected in bronchial epithelium of any of 10 control infants [6], [7]. Infant tissues were used as controls in these studies because they would be less likely to harbor a ubiquitous persistent or latent microbe than tissues from older children and adults, in the event that the KD agent is such a microbe. We examined acute KD ciliated bronchial epithelium using light and electron microscopy and showed that the antigen resides in intracytoplasmic inclusion bodies (ICI) that are consistent with aggregates of viral protein and nucleic acid [7].

We hypothesized that KD antigen detected in inclusion bodies during acute KD would be cleared by the immune system within the first two months after the onset of illness, when inflammation is most commonly observed in KD tissues [9]; we previously detected ICI in ciliated bronchial epithelium of 16/19 (84%) of acute KD patients but in 0/11 control infants [6], [7] (p<0.001). However, we recognized that ICI could be present in a small subset of older individuals with non-KD illness and of late-stage KD fatalities, because such individuals could be incidentally acutely infected or re-infected, respectively, with the KD ubiquitous respiratory microbe. If KD results from infection with a persistent or latent agent, the prevalence of ICI in KD tissues could be quite high, similar to that detected during acute infection. To determine the prevalence of ICI in these groups, we examined lung tissues from older children and adults with non-KD illness and tissues from KD patients who died ≥10 weeks after the onset of acute KD by light and electron microscopy.

## Results

We previously reported that intracytoplasmic inclusions (ICI) in acute KD ciliated bronchial epithelium were consistent with aggregates of viral proteins and associated nucleic acids [7]. To determine whether the ICI are manifestations of an acute infection that is cleared when acute signs of inflammation are resolved in KD (typically 8 weeks or less after the onset of fever [1]), we examined lung tissues from late-stage KD fatalities and controls. We also performed nucleic acid stains on ICI, to determine whether RNA or DNA was present.

### Immunohistochemistry (IHC)

**Figure 1:**
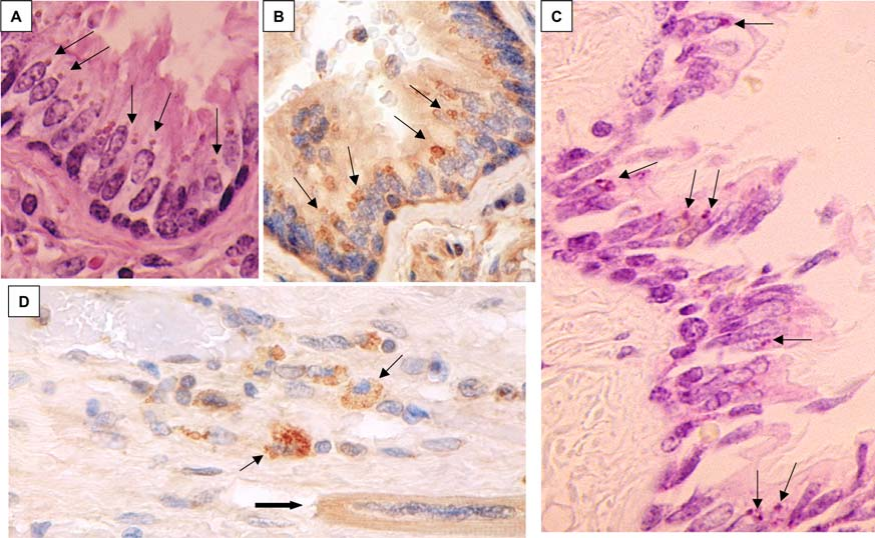
Light microscopic studies of tissues from KD patient 1. A, H&E of ciliated bronchial epithelium, demonstrating amphophilic supranuclear ICI (arrows); B, IHC using synthetic antibody J of ciliated bronchial epithelium, showing ICI (arrows); C, methyl green pyronin stain of ciliated bronchial epithelium, demonstrating red ICI (arrows) indicating the presence of RNA; D, IHC using synthetic antibody J on myocardium lesion, demonstrating antigen-positive macrophages (thin arrows) infiltrating an area that has undergone myocardial dropout near a vessel (thick arrow shows single viable cardiac myocyte in this field). A–D = 40X.

**Figure 2:**
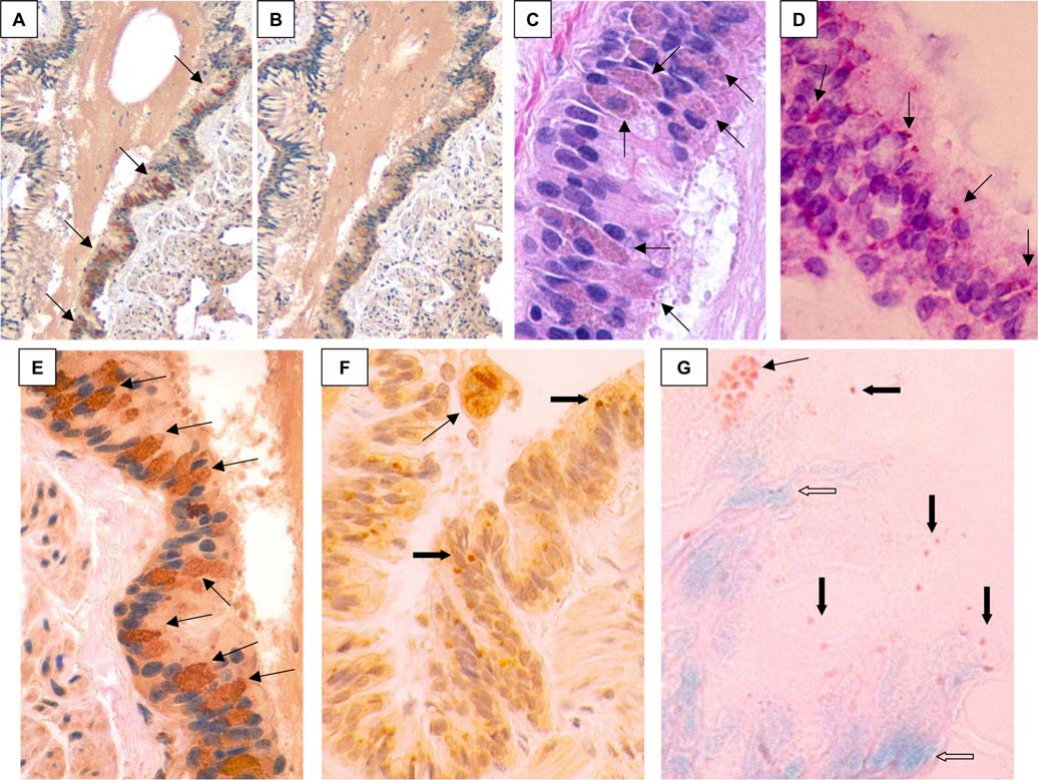
Light microscopic studies of ciliated bronchial epithelium from KD patient 3. A, immunohistochemistry (IHC) using synthetic antibody J, examples of specific staining of ICI indicated with arrows; B, IHC using control antibody I, no specific staining; C, H&E stain demonstrating that ICI are visible as amphophilic bodies (arrows); D, methyl green pyronin stain of paraffin section of a tangentially sectioned bronchus showing purple nuclei (DNA) and red ICI (RNA, arrows); E, higher-power image of Figure A, showing intense brown staining of multiple clusters of intracytoplasmic inclusion bodies (ICI, arrows); F, IHC from another bronchus, demonstrating fewer ICI (block arrows) and an IHC-positive macrophage in the lumen of the bronchus (thin arrow); G, frozen section of lung stained with methyl green pyronin; this unfixed tissue section shows typical blue-green nuclei (open arrows) and shows clusters of (thin arrow) and individual (black block arrows) ICI staining red, characteristic of RNA. A,B taken with 10X objective, E = 20X, C, D, F,G = 40X.

ICI were present in ciliated bronchial epithelium in 6/7 (86%) late-stage KD fatalities and 7/27 (26%) controls ages 9–84 years (p = 0.01). Dark brown-staining ICI, mostly supranuclear, were observed in ciliated bronchial epithelial cells from KD patients 1 (Figure 1B), 2, and 3 (Figure 2A, 2E, 2F) using synthetic antibody J; control antibody I gave negative results (Figure 2B) (Table 1). ICI were particularly numerous in KD patient 3; virtually all ciliated bronchioles had ICI. In KD patient 1, ICI were present in ciliated bronchioles in almost all blocks from different areas of the lung, but some bronchioles had many more ICI than others. In KD patient 2, ICI were less prevalent, with some bronchioles negative and others positive. In KD patients 4–6, ICI were less prevalent, with bronchi in some lung blocks entirely negative and in other lung blocks positive. ICI were not observed in available lung sections from KD patient 7, although ICI were observed in macrophages in peribronchial lymph node tissue in this patient.

**Figure 3:**
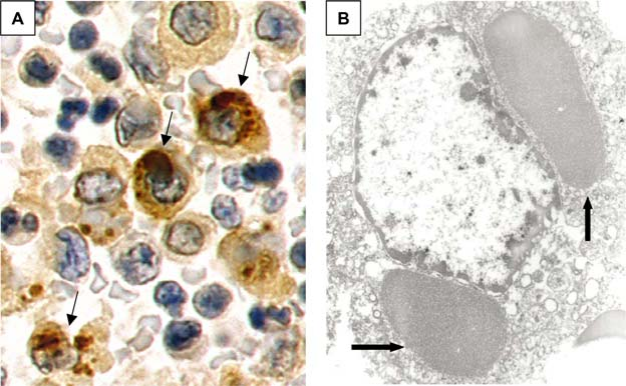
Peribronchial lymph node from KD patient 2. A, IHC using synthetic antibody J, showing antigen-positive macrophages containing spheroid bodies (arrows); B, transmission electron microscopy (TEM) of macrophage, showing two large, finely granular ICI (arrows). A = 40X, B = 20,000X.

**Table 1:** Patients with a history of Kawasaki Disease (KD) and the pathologic findings in their lungs.

Patient	Time since KD onset	Cause of death	H&E	IHC	MGP/Feulgen	TEM
1 (F, 8 yrs, Asian)	15 months	Ventricular tachycardia	Heart failure	+	+/−(epithelium)	ICI
2 (M, 19 mos, Black)	9 months	Congestive heart failure	ARDS, PE	+	+/−(macrophage)	ICI
3 (M, 24 yrs, Hispanic)	21 years	Head trauma	Heart failure, PE	+	+/−(epithelium)	ICI
4 (NA, NA, Japanese)	14 months	Bronchiolitis	Bronchitis, atelectasis	+	ND	ND
5 (M, 5 mos, Japanese)	11 weeks	Myocardial infarction	ARDS	+	ND	ND
6 (F, 4 yrs, Japanese)	10 weeks	Status asthmaticus	No pathology	+	ND	ND
7 (F, 14 mos, Japanese)	8 months	Myocardial infarction	Heart failure	−	ND	ND

NA = not available, ND = not done, PE = pulmonary emboli, ARDS = acute respiratory distress syndrome, IHC = +indicates staining with antibody J but not antibody I,-indicates negative results with I and J

In KD patients 1–6, as in the KD patients reported in our prior study (6,7) there was a striking restriction in the distribution of ICI in bronchi. ICI were not present in large bronchi completely surrounded by cartilage, but were predominately localized to the ciliated cells of the terminal or tertiary bronchi and especially the proximal bronchioles, where cartilage is absent and relatively few goblet cells are present. ICI extended to ciliated terminal bronchioles lacking goblet cells, but were not present in more distal bronchioles that lacked cilia. A subset of lung macrophages, usually peribronchial, in patients 1, 2, and 3 were positive. ICI were observed in macrophages in peribronchial lymph nodes in patients 2, 5 and 7; in patient 2 they appeared globular (Figure 3A). Occasionally, an antigen-positive macrophage could be observed in the lumen of a positive bronchus (Figure 2F), although most luminal macrophages were antigen-negative. Myocardium was available for study from patients 1 and 2; antigen-positive macrophages infiltrating areas of myocardial dropout near blood vessels were observed in the myocardium of patient 1 (Figure 1D). Antigen-positive macrophages were also present in the adventitia of some small coronary arteries within the myocardium. ICI were observed in ciliated bronchial epithelial cells in 7 of 27 controls ages 9–84 (Table 2). ICI in control lung were localized predominately to proximal bronchioles, as in the KD patients. Intranuclear inclusion bodies were never observed in KD patients or controls.

**Table 2:** Controls and the pathologic findings in their lungs.

Control	Cause of death or underlying illness	H&E	IHC
1 (83 years, F)	Cerebrovascular accident, diabetes	Heart failure	+
2 (76 years, M)	Cerebrovascular accident	COPD, pneumonia	+
3 (22 years, F)	Meningioma, renal failure, urosepsis	Heart failure, bronchitis	+
4 (18 years, F)	Renal transplant, myocardial infarction	Heart failure, bronchitis	+
5 (59 years, M)	Non-small cell carcinoma	COPD, heart failure, atelectasis	+
6 (16 years, M)	Heart transplant, coronary vasculopathy	COPD, heart failure	+
7 (71 years, F)	Squamous cell carcinoma	Heart failure, atelectasis	+
8 (81 years, F)	Cerebrovascular accident	COPD	-
9 (61 years, M)	Adenocarcinoma	Heart failure	-
10 (69 years, F)	Squamous cell carcinoma	Heart failure	-
11 (52 years, F)	Emphysema	COPD, heart failure	-
12 (66 years, M)	Adenocarcinoma	Normal	-
13 (37 years, F)	Adenocarcinoma	Heart failure	-
14 (NA, M)	Adenocarcinoma	Heart failure	-
15 (63 years, M)	Poorly differentiated adenocarcinoma	Heart failure, hemorrhage	-
16 (48 years, M)	Emphysema	Heart failure	-
17 (73 years, M)	Myocardial infarction	Normal	-
18 (63 years, M)	Cardiac arrest, diabetes	Edema	-
19 (79 years, M)	Myocardial infarction, Parkinson's disease	COPD, heart failure	-
20 (84 years, F)	Peripheral vascular disease	COPD, heart failure	-
21 (76 years, M)	Cardiac failure	COPD, heart failure	-
22 (66 years, M)	Intracranial hemorrhage, kidney transplant	Heart failure, pneumonitis	-
23 (14 years, F)	Truncus arteriosus, GI bleed	Pneumonia, edema, heart failure	-
24 (17 years, F)	SLE, multiorgan dysfunction, shock,	Heart failure	-
25 (9 years, M)	Medulloblastoma, stem cell transplant	Septic emboli, infarcts	-
26 25 (67 years, M)	Squamous cell carcinoma	Edema, hemorrhage, heart failure	-
27 (79 years, M)	Adenocarcinoma	COPD, heart failure, bronchitis	-

NA = not available, COPD = chronic obstructive pulmonary disease, SLE = systemic lupus erythematosis, IHC = +indicates staining with antibody J but not antibody I,-indicates negative results with I and J

### Hematoxylin-eosin (H&E)

The lung from KD patient 1 showed evidence of acute heart failure in the form of fresh RBCs in alveolar spaces. There was also focal atelectasis and fibrin thrombi in scattered capillaries, indicative of diffuse intravascular coagulopathy (DIC). Changes consistent with adult respiratory distress syndrome (ARDS) referred to by pathologists as diffuse alveolar damage (DAD) were observed in the lung from KD patient 2, along with pulmonary emboli without infarcts, and mild chronic bronchitis. Lung from KD patient 3 showed evidence of chronic heart failure in the form of abundant hemosiderin-laden macrophages in alveolar spaces. There was also focal atelectasis and organizing pulmonary emboli, but no infarcts. ICI were easily visualized in H&E-stained sections as supranuclear amphophilic bodies in bronchioles from KD patient 1 (Figure 1A) and 3 (Figure 2C). Most bronchioles containing ICI were otherwise normal in appearance (Figures 1A–C, Figures 2A–F). However, shedding epithelial cells was a characteristic feature of occasional bronchioles. Lung from KD patient 4 showed bronchiolitis with atelectasis and edema, and from KD patient 5 showed ARDS and atelectasis. Lung from KD patient 6 appeared normal, and from KD patient 7 revealed changes consistent with heart failure (Table 1). Findings in the lungs of controls are indicated in Table 2.

### Nucleic acid stains

The Feulgen stain, which identifies DNA, did not visualize ICI in bronchial epithelial cells in KD patients 1 or 3. However, methyl green pyronin (MGP), which stains RNA red, stained ICI red in bronchioles from both patients (Figures 1C, 2D, 2G). The cytoplasm of plasma cells in the subepithelial tissues and nucleoli also stained red and served as internal controls for RNA in the MGP preparations. In frozen sections (available only for patient 3), nuclei stained blue and ICI were red, again indicating the presence of RNA and not DNA in ICI (Figure 2G). In peribronchial lymph node of KD patient 2, MGP stains revealed red spheroids within scattered macrophages; cytoplasm of plasma cells served as an internal control for RNA and also stained red. Nucleic acid stains were not performed on tissues from patients 4–7, because fewer ICI were present.

### Transmission electron microscopy (TEM)

**Figure 4:**
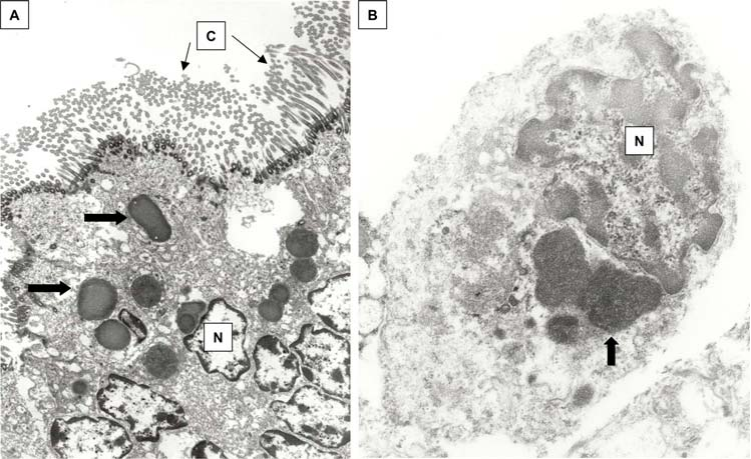
TEM of formalin-fixed, paraffin-embedded lung from KD patient 1. A, ciliated bronchial epithelium demonstrating electron-dense apical ICI (block arrows). B, alveolar macrophage, demonstrating perinuclear, finely granular spheroid bodies similar to those seen within the bronchial epithelium (block arrow). N = nucleus, C = cilia. A = 9,500X, B = 26,000X.

Formalin-fixed, paraffin-embedded lung sections from KD patient 1 revealed numerous supranuclear, electron-dense, granular ICI in ciliated bronchial epithelial cells (Figure 4A). Rare alveolar macrophages in KD patient 1 were noted to have perinuclear, finely granular rounded bodies similar to those seen within the bronchial epithelium (Figure 4B), and occasional macrophages in peribronchial lymph nodes from KD patient 2 contained large finely granular ICI (Figure 3B).

**Figure 5:**
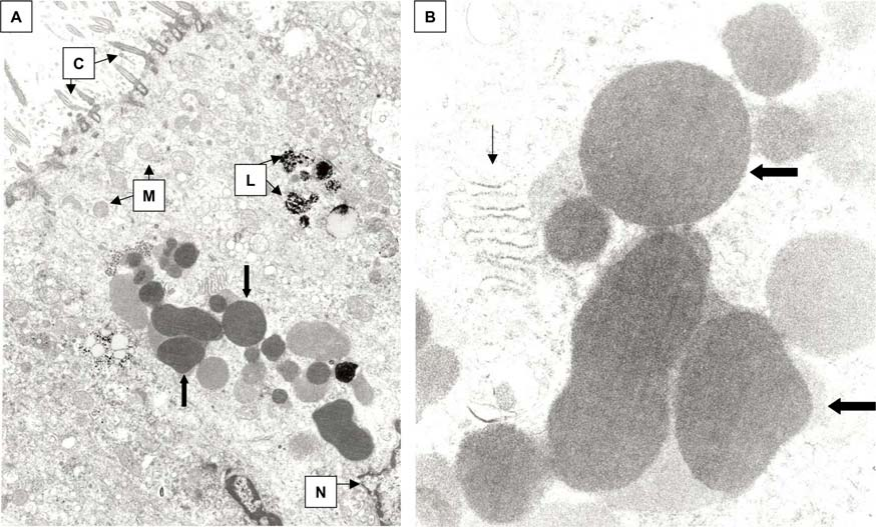
Transmission electron microscopy (TEM) of ciliated bronchial epithelium from glutaraldehyde-fixed, previously frozen lung from KD patient 3. A) Lower power image demonstrating finely granular, variably electron-dense ICI (block arrows). N = nucleus, L = lysosomes, M = mitochondria, C = cilia. B) Higher power image of ICI (block arrows). Note rough endoplasmic reticulum (thin arrow). ICI are spherical and do not appear membrane-bound. A = 14,000X, B = 52,000X.

Frozen lung from KD patient 3 placed into glutaraldehyde allowed for improved visualization of intracellular structures compared with tissue previously embedded in paraffin from the same patient. Rough endoplasmic reticulum, mitochondria, lysosomes and nuclei were all well visualized in glutaraldehyde-fixed ciliated bronchial epithelial cells (Figure 5A). Numerous ICI of varying electron-density, likely corresponding to the concentration of the protein and nucleic acid contents, were observed predominantly in a supranuclear location in KD patient 3 (Figure 5A); higher-power views indicated that ICI were mostly granular and smooth surfaced and did not appear to be membrane-bound (Figure 5B). However, fine detail was lost during freezing; for example, it was not possible to determine whether or not ICI represented aggregates of nucleocapsids. No viral particles or other microbial elements were identified in any cells, including those containing ICI.

No intranuclear inclusion bodies were observed by TEM in KD patients 1, 2, or 3.

## Discussion

We previously reported that KD synthetic antibodies detect antigen in ciliated bronchial epithelium of acute KD patients (75% of these patients were infants), but not of infant controls [6], [7]. The antigen was not any of 40 common inflammatory proteins [6], and further study indicated that the antigen was localized to ICI that appeared consistent with aggregates of viral protein and nucleic acid [7]. We initially hypothesized that ICI were present only during acute KD and were subsequently cleared by the immune system. However, in the present study, KD antigen was detected in ICI in ciliated bronchial epithelium in 6 of 7 (86%) late-stage KD fatalities and in 7 of 27 (26%) older childhood or adult controls (p = 0.01). The very high rate of detection of ICI in late-stage KD fatalities is strongly suggestive of establishment of persistent or latent infection following the acute illness. The detection of ICI in 86% of late-stage KD fatalities is virtually identical to the 84% rate of detection (16/19) of ICI in our two combined previous studies of KD fatalities in the first two months after the onset of illness [6], [7]. It is possible that an even higher percentage of KD patients harbor ICI in ciliated bronchial epithelium, but that insufficient archival tissue was available from some patients to demonstrate the ICI. In several KD patients in whom an initially tested lung section did not reveal ICI, examination of sections from additional lung blocks clearly showed ICI. Because epidemiologic data are consistent with a ubiquitous agent as the cause of KD [1], with frequent asymptomatic or mildly symptomatic infection in the general population, establishment of a persistent infection by the agent in some individuals would likely result in the detection of ICI in a subset of older children and adult controls, consistent with our findings. Although re-infection is a possibility, we believe that persistent infection is more likely, in view of the high rate of ICI detection in late-stage KD fatalities and because acute infection in one-quarter of a group of random controls seems unlikely even for a ubiquitous infectious agent.

It is notable that in the classic 1980 study by Amano and colleagues on the general pathology of acute KD, inflammatory lesions were unusual in most organs later than 60 days after the onset of fever, but “in the lung, spleen, salivary gland or lymph nodes, inflammation was considered to be persistent or recurrent [9].” The authors studied tissues from 9 KD children who died after the 60^th^ day of illness, with death ranging from 66 days to 4.5 years after the onset; 7/9 had evidence of bronchitis. Antigen was not detected in either of 2 salivary gland tissues available to us from acute KD fatalities (data not shown); we have previously reported positive results of antigen detection in macrophages in lymph nodes and spleen in children with acute KD [6], and in this study we report antigen detection in the lung of KD patients months to years after the acute illness.

Several RNA viruses, most notably the human and animal paramyxoviruses, have been associated with persistence in infected cells. Persistence of cytoplasmic and nuclear inclusion bodies in human brain was demonstrated in subacute sclerosing panencephalitis (SSPE), a persistent measles virus infection. Canine distemper virus can result in persistence of ICI in tissue culture cells [10]. Randall and colleagues showed that simian virus 5 (SV5) can establish persistent infection by remaining inactive in ICI [11]–[13]. In contrast to measles virus in SSPE, infectious SV5 virus occasionally can be reactivated from ICI in persistent infection [11]. It has been proposed by these authors that some paramyxoviruses may form ICI as a viral defense mechanism to evade the immune system [13]. In this model, when the virus is exposed to interferon, it halts production of its glycoproteins, which are no longer expressed on the surface of infected cells, and viral nucleocapsid proteins accumulate in ICI [13]. Because viral glycoproteins are not expressed on the cell surface, the immune system does not recognize the cell as infected. The virus can remain inactive within ICI for prolonged periods of time but occasionally can be reactivated and viral replication reinitiated [12]. The infected host could then release virus into the environment until a secondary immune response once again forces the virus to recede back into inactive ICI.

At least 20 cases of Kawasaki Disease-like illness have been reported in HIV-infected adults [14]. In these patients, moderate to severe immune dysfunction and high viral loads have been characteristic [14]. This has led some authors to propose that these patients are experiencing reactivation of a persistent infectious agent of KD following childhood infection [15]. A persistent infectious agent could also explain recurrences of KD in at least 1–3% of affected children [1].

Bronchial epithelial cells undergo turnover when injured, and therefore, cells containing ICI could be destroyed or shed. However, our hypothesis is that newly formed ciliated bronchial epithelial cells could become infected during reactivation of the putative viral agent in adjacent cells containing ICI; the agent would have a short time period to replicate and be shed before memory immune responses would force it to recede back into inclusion bodies. The clinical illness of KD would only recur in those individuals in whom memory immune responses were not adequate to prevent systemic spread of infection. Individuals in whom ICI form and persist could intermittently shed infectious virus over a long period of time [12]; this would be a very effective way for a ubiquitous respiratory virus to spread through the population. Healthy asymptomatic individuals in isolation at the South Pole have been reported to intermittently shed parainfluenza virus over a 6–8 month period, with two outbreaks of respiratory illness occurring within that 8 month period best explained as being initiated by persistently infected individuals [16]. Lung tissue was not available from persistently infected individuals in this study, and it is not known whether these human parainfluenza viruses formed ICI in bronchial epithelium. Other viruses such as human metapneumovirus and rhinovirus have been reported to result in persistent infection in immunocompromised hosts [17], [18]. Viral persistence related to the formation of ICI has not been demonstrated in these studies, and clearly the KD agent is different from the known human respiratory viruses.

Our findings emphasize the likelihood that macrophages play a key role in the pathogenesis of KD. It is not clear whether antigen-positive macrophages in KD patients are scavengers and/or whether they become infected with the KD agent. Macrophages could be responsible for uptake of the agent from bronchioles and delivery to other distal sites, such as the coronary arteries, as they circulate through the bloodstream and into tissues. To date, we have not observed antigen-positive lymphocytes or neutrophils in acute or late-stage KD patient tissues.

ICI in KD patients and controls were strikingly localized to the ciliated epithelial cells of smaller bronchi and bronchioles. ICI were not present in non-ciliated cells or in ciliated cells of trachea or large bronchi completely surrounded by cartilage. This remarkable restriction in localization of ICI leads us to speculate that only the ciliated epithelial cells lining the smaller bronchi and bronchioles express a receptor that facilitates entry of the KD agent into the cell or a cellular gene that facilitates formation of ICI in these cells.

We are utilizing various methods to identify the proteins and nucleic acids within the ICI, and we believe that this information will likely lead to the identification of the KD etiologic agent. These efforts have been hampered by the extreme scarcity of fresh tissue samples from KD fatalities. Several studies in which RNA extracted from KD tissue has been analyzed on viral microarrays have so far not yielded a positive result (data not shown). We believe it likely that the KD agent is a currently unknown RNA virus that may not show significant homology to known viruses. Efforts to obtain fresh lung and coronary artery tissue from KD fatalities for molecular studies and for direct placement into glutaraldehyde for optimal ultrastructural experiments should continue. In addition, our data indicate that antigen-positive unfixed tissue from adult controls could potentially be utilized in these experiments.

In conclusion, we report that ICI are present in ciliated bronchial epithelial cells in about 85% of KD patients who died months to years after the acute illness, that the antigen detected by KD synthetic antibody and identified within ICI in ciliated bronchial epithelial cells could also be identified in a subset of macrophages in lung, peribronchial lymph nodes, and damaged myocardium from some KD patients months to years after acute KD, that the nucleic acid within these ICI is RNA and not DNA, and that ICI can be observed in ciliated bronchial epithelium in about 25% of controls ages 9–84. These findings provide new directions in the search for the etiologic agent of KD, and point to a ubiquitous, previously unrecognized RNA virus that can result in persistent infection as the causative agent.

## Materials and Methods

### Patients and Specimens

Formalin-fixed, paraffin-embedded lung tissue was available for light microscopy from all of 7 late-stage KD fatalities (in whom death occurred ≥10 weeks after the onset of fever) and 27 controls. Three KD patients died of myocardial infarction or congestive heart failure as a result of coronary artery aneurysms, one died postoperatively following a procedure to resect giant aneurysms, and three died of causes unrelated to KD (Table 1). Selected other tissues were available for study from KD patients 1 and 2, such as myocardium and lymph node.

Patient 1 developed KD at age 7 years. She was initially treated with antibiotics, and KD was diagnosed when echocardiography showed a large coronary artery aneurysm. She was treated with two infusions of IVIG and maintained on long-term coumadin and aspirin. Fifteen months after the onset of illness, she underwent elective resection of bilateral giant coronary artery aneurysms with homograft placement, but died two hours postoperatively following the development of hypotension and ventricular tachycardia. The ethnicity of patient 1 was described as “Asian”, but further information was not available.

Patient 2 was admitted at 19 months of age with cough and congestion for one week without fever; rales were detected on examination of the chest. The clinical diagnosis was possible myocarditis or endocarditis. Despite intensive care, he suffered multiple cardiac arrests and died 3 days after admission. Autopsy revealed severe congestive heart failure, dilated coronary arteries, necrotizing arteritis of all the coronary arteries with occlusions, and areas of myocardial infarction. In retrospect, the child had had a febrile illness at 10 months of age that was diagnosed as a streptococcal infection, but likely represented an episode of KD.

Frozen lung tissue from KD patient 3 was provided by the National Disease Research Interchange (NDRI). Patient 3, a 24-year-old male, had KD at age 3. Because of confidentiality issues, it was not possible to obtain further history regarding the KD illness. The patient had Wolff-Parkinson-White syndrome and underwent an ablation procedure 7 years prior to death. The patient was found underneath a car with severe head trauma, and toxicology studies were positive for cocaine and phencyclidine (PCP). He was reported to be well prior to this event. His heart grossly appeared normal, and was used for transplant. One lung was used for transplant, and frozen tissue from the other lung was available for research. Serologic studies for HIV, syphilis, hepatitis B and hepatitis C infection were negative. Information regarding the condition of the recipient(s) of the other lung and heart was not available to NDRI because of confidentiality issues.

Patients 4 died of acute bronchitis and patient 6 died of asthma. Patients 5 and 7 died of myocardial infarction as a late consequence of KD (Table 1).

Control tissues were obtained from grossly normal-appearing areas of lung at the time of lung transplantation, lung biopsy, or autopsy. Lung tissue from controls 1,2, 8,9,17–19, and 20–22 were provided by NDRI. For controls, information regarding a past history of KD, a prolonged febrile illness during childhood, and/or the degree of immunosuppression administered to the cancer and transplant patients was not available.

Frozen lung tissue from KD patient 3 (Table 1) was partially thawed, bronchi were dissected, and the tissue was placed in glutaraldehyde for examination by TEM. Formalin-fixed, paraffin-embedded lung sections and blocks from KD patient 1 (Table 1) were studied by TEM. The present study was approved by the Institutional Review Boards of Children's Memorial Hospital and Loyola University Medical Center.

### Synthetic KD antibodies

Synthetic KD antibodies were made as previously described [6]. IHC was performed with synthetic antibodies J and I [7]. Antibody J shows strong binding to acute KD but not to control infant ciliated bronchial epithelium; control antibody I does not demonstrate binding [6], [7], [8].

### Immunohistochemistry

Formalin-fixed, paraffin-embedded tissue sections were deparaffinized by use of xylene, rehydrated, and heated in 10 mM sodium citrate buffer (pH 6.0), to enhance antigen retrieval [6]. Sections were incubated with 10–15 micrograms/ml biotinylated synthetic antibody J or I and color developed using the Vectastain Elite ABC kit (Vector). Diaminobenzidine tetrahydrochloride was used as a reaction product, to generate a brown stain. Sections were lightly counterstained with hematoxylin. We recorded positive results when strong brown staining of ICI was observed in ciliated bronchial epithelial cells, or in tissue macrophages.

### Hematoxylin-eosin, methyl green pyronin (MGP), and Feulgen stains

Nucleic acid stains were performed on tissues from KD patients 1 and 3, which were strongly positive for ICI. Standard hematoxylin-eosin, MGP, and Feulgen staining was performed on formalin-fixed, paraffin-embedded lung tissues. MGP staining was also performed on frozen sections of lung from KD patient 3, and formalin-fixed peribronchial lymph node from KD patient 2.

### Transmission electron microscopy (TEM)

TEM was performed on tissues from KD patients 1 and 3, because ICI were especially abundant in these patients. TEM was performed in 2 different laboratories. At Loyola University, resin blocks were made from formalin-fixed, paraffin-embedded tissue sections from KD patients 1 and 3 after IHC with synthetic antibody, to allow for accurate localization of ICI within bronchi, as previously described [7]. At George Washington University, pieces of formalin-fixed, paraffin-embedded tissues were excised directly from areas of tissue blocks that were positive by IHC, as previously described [7]. In addition, TEM was performed on frozen lung from KD patient 3 that was placed into glutaraldehyde, as described above.

### Statistical analysis

The prevalence of ICI in KD patients and controls was compared using a two-tailed Fisher's exact test, with a p≤0.05 considered significant.
